# Self-Inflicted

**Published:** 1875-05

**Authors:** 


### SELF-INFLICTED.

Our lady readers may not thank us for intimating, quite plainly, that the diseases from which they suffer, and which are peculiar to females, are in the great majority of instances the result of their own imprudence and folly. But it is a fact, an unpleasant fact, yet one not at all difficult to prove. The physician who would tell a patient that she had a serious disease, but it was all her own fault, would be considered rather rude and unsympathising. And yet if asked concerning the cause of her troubles he would refer it to certain practices which were voluntary on the part of the patient. This would be milder and more acceptable language, with the import not at all modified.

The writer proposes to allude to one or two of the causes of those diseases and "weaknesses" so prevalent among women :

First, the wearing of corsets. It can hardly be called a garment, but the corset seems, from its universal use, to be regarded as an indispensable article of dress. It is often an instrument of torture ; it is always a disease-producing contrivance. The woman who wears one is always relieved when, after making a round of calls or taking a walk in the streets, she gets home and loosens the laces. Perhaps she has suffered from shortness of breath, or from palpitation of the heart, and she finds those unpleasant symptoms immediately disappear. The exercise of walking excites the circulation, increases the action of the heart and hurries the respiration. All this is natural, and because natural, healthy. (We mean, of course, within due limits.) But with the forced compression of the chest by the corset, the lungs are unable to expand perfectly and freely, and the heart is seriously trammeled in its action. Both these organs are required to act more vigorously, in short, do more work, than when the body is at rest. And both are bound and fettered. No wonder that palpitation, shortness of breath and pain in the side should result. It would seem a very foolish thing if a lady, by some device in dress, should interfere with the movements of her limbs as much as she does with those of the chest and the vital organs which it contains.

The heart and lungs are not the only organs which suffer from corsets. All the contents of the body below the diaphragm are subjected to a downward pressure, and, in the course of a short time, are more or less forced from their natural positions. But the uterus is the organ which is affected to the greatest extent. When we consider that ninety-nine women in every hundred wear corsets, and that ninety-eight of them draw their laces so tightly as to be uncomfortable, we cannot be surprised that displacements of the uterus are now so very prevalent. It cannot be otherwise. For several hours each day there is a constant pressure exerted for the downward displacement of the abdominal contents. Sooner or later the effect of this pressure is felt. The uterus more than any other is forced out of place. Its yielding supports cannot resist the continuous pressure from above. As an illustration of the direct effect of tight lacing in causing displacements, we refer to a late writer who declares that he has been unable, in some cases, to return the organ to its normal position until the clothes were loosened.

Another direct result of tight lacing is the obstruction of the circulation.

The return of the blood from the pelvic viscera is greatly impeded, inducing congestion of the uterus and other organs. Inflammation may follow; enlargement of the organ with increased weight is sure to result. Then comes the pain in the back, the general discomfort and special "bearing down" distress.

Another cause of uterine disease is the violence to which that organ is subjected in order to prevent its discharging its natural functions. We need use no more specific language for every woman, especially every guilty and suffering one, will understand our meaning. In these days it is not the bearing and nursing of children that ruins the health of so many women. It is rather the unceasing war against nature during so many years of the best part of the woman's life; it is, in short, her persistent efforts to thwart the designs of Him to whom she owes her creation and preservation. She is often successful, but her success is followed by fearful penalties.

The two practices to which we have made allusion are very frequent causes of uterine troubles. It may not be too much to say that the great majority of female diseases may be traced directly to one or the other, and often to both of these vicious practices.

Therefore the verdict is a just one, which throws upon the sufferer the responsibility and *the guilt* of producing in her own body a host of ills which unfit her for the duties of life and perhaps make her a burden to herself and her friends. But the mischief being done, we must inquire what are the most available means of cure. On this subject we shall, in our next number, offer a few suggestions under the head of *Self-Remedied.*

---

You must be clean to be healthy.

